# Microbiota, Immune Subversion, and Chronic Inflammation

**DOI:** 10.3389/fimmu.2017.00255

**Published:** 2017-03-13

**Authors:** Carolyn D. Kramer, Caroline Attardo Genco

**Affiliations:** ^1^Department of Integrative Physiology and Pathobiology, Tufts University School of Medicine, Boston, MA, USA

**Keywords:** microbiota, inflammation, toll-like receptors, innate immunity, immune subversion, immune dysregulation, atherosclerosis

## Abstract

Several host-adapted pathogens and commensals have evolved mechanisms to evade the host innate immune system inducing a state of low-grade inflammation. Epidemiological studies have also documented the association of a subset of these microorganisms with chronic inflammatory disorders. In this review, we summarize recent studies demonstrating the role of the microbiota in chronic inflammatory diseases and discuss how specific microorganisms subvert or inhibit protective signaling normally induced by toll-like receptors (TLRs). We highlight our work on the oral pathogen *Porphyromonas gingivalis* and discuss the role of microbial modulation of lipid A structures in evasion of TLR4 signaling and resulting systemic immunopathology associated with atherosclerosis. *P. gingivalis* intrinsically expresses underacylated lipid A moieties and can modify the phosphorylation of lipid A, leading to altered TLR4 signaling. Using *P. gingivalis* mutant strains expressing distinct lipid A moieties, we demonstrated that expression of antagonist lipid A was associated with *P. gingivalis*-mediated systemic inflammation and immunopathology, whereas strains expressing agonist lipid A exhibited modest systemic inflammation. Likewise, mice deficient in TLR4 were more susceptible to vascular inflammation after oral infection with *P. gingivalis* wild-type strain compared to mice possessing functional TLR4. Collectively, our studies support a role for *P. gingivalis*-mediated dysregulation of innate and adaptive responses resulting in immunopathology and systemic inflammation. We propose that anti-TLR4 interventions must be designed with caution, given the balance between the protective and destructive roles of TLR signaling in response to microbiota and associated immunopathologies.

## Introduction

Many inflammatory conditions and immunological disorders have been recently linked to the microbiota (1). Studies in humans have documented that both shifts in the microbiota (dysbiosis) and specific microorganisms are associated with these immunological disorders (2–4). A number of epidemiological studies have reported phylogenetic differences in the presence and relative abundance of specific microbial communities between subjects with a particular disease and “healthy” individuals (5–7). While overall shifts in biodiversity within (alpha diversity) or among (beta diversity) subject samples are often reported, more recent work has considered functional diversity elucidated by metagenomic analyses (8, 9). The most well-studied microbial dysbiosis is that of the gut microbiota, which is associated with inflammatory bowel diseases (IBD) and colorectal cancer (7, 9–11). Dysbiosis of the oral microbiota has been associated with oral squamous cell carcinoma (OSCC), and dysbiosis of the lung microbiota has been associated with cystic fibrosis (CF) (12–15). However, gut microbiota dysbiosis has also been associated with non-intestinal diseases including obesity, type 1 diabetes (T1D), rheumatoid arthritis (RA), and atherosclerosis (16, 17). Likewise, dysbiosis of the oral microbiota has been associated with diseases occurring outside of the oral cavity, such as lung and pancreatic cancers as well as atherosclerosis and RA (18–21).

In addition to dysbiosis, the presence of specific microorganisms has also been associated with cancer, atherosclerosis, autoimmune disorders, and RA (22–40). Indeed, much of the experimental evidence aimed at defining mechanistic links between the microbiota and systemic inflammatory conditions has focused on metabolic and immunological pathways induced by specific microorganisms. In this review, we summarize recent studies aimed at defining immunological mechanisms that link specific microorganisms to low-grade chronic inflammation and immunopathology.

## Microbiota and Chronic Inflammatory Diseases

In June 2012, the Human Microbiome Project Consortium (HMP) reported on the “healthy” microbiome at 15–18 anatomic sites and provided a framework for future studies on defining the association of the microbiome with disease states. In the few years since the healthy baseline was established, a number of reports have defined altered microbiota that may contribute to disease. A common finding among studies investigating dysbiosis of the microbiota was a decrease in alpha diversity in diseased vs. healthy states (41–48). Many studies have also demonstrated that changes in the microbiome correlate with the pathogenesis of various systemic inflammatory diseases (17, 18, 47, 48). As might be expected, changes in the microbiota specific to a particular anatomic location have been associated with inflammatory diseases of that area as well as distant tissues or organs.

### Association of Microbiota with Local Inflammation

Several studies have linked IBD with dysbiosis of the gut microbiota characterized by decreases in *Bifidobacterium, Clostridium*, and *Faecalibacterium prausnitzii* and increases in *Ruminococcus gnavus* and adherent-invasive *Escherichia coli* (46, 49–51). In subjects with IBD, there was also a decrease in the genus *Roseburia*; interestingly, this decrease was observed in healthy subjects with a high genetic risk for IBD (52–55). Not only was dysbiosis of the gut microbiota associated with IBD but distinct shifts in the gut microbiota and a decrease in alpha diversity were also shown to distinguish ileal vs. colonic Crohn’s disease (55). Significant phylogenetic differences were found between patients who respond to treatment for ulcerative colitis vs. those who do not respond to treatment (53).

Many cancers have now been linked to dysbiosis of the local microbiota. The gut microbiome of colorectal cancer patients includes significantly higher populations of *Enterococcus faecalis, Streptococcus bovis*, and *Fusobacterium* than the microbiota of healthy controls (56). The oral cancer OSCC is associated with a shift in the oral microbiome. *Streptococcus* species dominate the salivary microbiota within OSCC tumor sites compared to non-tumor sites within the same individual (57). There also is significant enrichment of Actinobacteria, Bacteroidetes, Firmicutes, Proteobacteria, *Porphyromonas*, and *Treponema* in the uterine microbiota of individuals with endometrial cancer (58).

### Association of Microbiota with Systemic Inflammation at Sites Distant from Infection

Changes in the gut microflora have also been associated with metabolic and inflammatory conditions distant from infection including obesity, diabetes, and autoimmune diseases. Studies have reported that the relative proportion of Actinobacteria and Bacteroidetes was increased and decreased, respectively, in the gut microbiota of obese individuals compared to lean individuals (43, 59). An increased abundance of Bacteroidetes and *Bacteroides* and a decreased abundance of Firmicutes and *Bifidobacterium* and *Prevotella* were also reported to distinguish the intestinal microbiome of children with T1D from that of age-matched healthy controls (45, 60–62).

An increased abundance of *Prevotella* and a decreased abundance of *Bacteroides* were reported in stool samples from subjects with new-onset RA compared to healthy controls (63). A separate study characterized an RA-associated fecal microbiome as one in which Actinobacteria and *Collinsella* and *Eggerthella* were consistently expanded while *Faecalibacterium* was notably decreased compared to healthy controls (47). A recent study examined both the gut and oral microbiota of subjects with RA and reported increased levels of *Lactobacillus salivarius* and decreased levels of *Haemophilus* spp. compared to healthy controls in both sites (64). Shifts in the oral microbiome have been reported in subjects with RA, including lower levels of *Corynebacterium* and *Streptococcus* compared to healthy controls (18). Subjects with new-onset RA also have shifts in the oral microbiota with increases in *Prevotella* and *Leptotrichia;* these organisms were absent in the oral microbiota of healthy controls (18). Bronchoalveolar lavage fluid from subjects with early RA revealed dysbiosis of the lung microbiome that was attributed to a significant decrease in *Actynomyces, Burkholderia, Porphyromonas, Prevotella*, and *Treponema* compared to healthy controls (48).

Some cancers have been associated with oral microbiota changes at sites distant from the primary tumor. In saliva samples from subjects with lung cancer, *Capnocytophaga, Selenomonas*, and *Veillonella* were more abundant and *Neisseria* was less abundant compared to healthy controls (19). The presence of the oral pathogens *P. gingivalis* and *Aggregatibacter actinomycetemcomitans* in oral wash samples were associated with a higher risk of pancreatic cancer, while presence of Fusobacteria and *Leptotrichia* in oral wash samples was associated with a lower risk of pancreatic cancer risk (65).

Shifts in the gut and oral microbiota have also been associated with symptomatic atherosclerosis patients. The gut microbiota of subjects with atherosclerosis had increased levels of *Desulfovibrio, Enterobacter, Megasphaera*, and *Oscillibacter* and less *Bacteroides, Faecalibacterium*, and *Prevotella* compared to asymptomatic subjects (66). Subjects with symptomatic atherosclerosis have elevated levels of several genera of bacteria in the oral cavity, including *Anaeroglobus* and *Porphyromonas* (20).

## Innate Immune Mechanisms Linking Specific Microorganisms to Chronic Inflammation and Immunopathology

A number of studies have examined the ability of pathogens to induce systemic inflammation and immunopathology at sites distant from infection. Well-defined animal models of RA, cancer, and atherosclerosis have been utilized to demonstrate a link between infection with specific pathogens and acceleration of disease (67–75). Many of these studies have focused on the role of the innate immune system and in particular toll-like receptors (TLRs) in microbial-induced immunopathology and disease (70, 73, 76–82).

Toll-like receptors detect conserved microbial products and play a central role in the activation of innate and adaptive immune pathways (83, 84). TLR2 and TLR4 are two of the most well-characterized TLRs that respond to microbial membrane components. TLR2 is a cell-surface receptor that recognizes pathogen-associated molecular patterns (PAMPs) that are typically associated with both Gram-positive and Gram-negative bacteria, such as lipoproteins, lipoteichoic acid, peptidoglycan, zymosan, and porins (85–89). TLR4 is a cell-surface receptor that recognizes lipopolysaccharide (LPS) from Gram-negative bacteria (90–92). Signal transduction following recognition of LPS by the TLR4 complex (CD14–TLR4–MD2) is an essential component of host immunity to Gram-negative bacterial infection (93). TLR4 signals through MyD88- and TRIF-dependent pathways to promote proinflammatory cytokine production and type I IFN (IFNβ) responses, respectively (94, 95). In addition to PAMPs, various endogenous “danger” molecules released from damaged host cells activate TLRs; these molecules are known as danger-associated molecular patterns and include heat-shock proteins, hyaluronic acid, and oxidized low-density lipoprotein (96).

**Figure 1:**
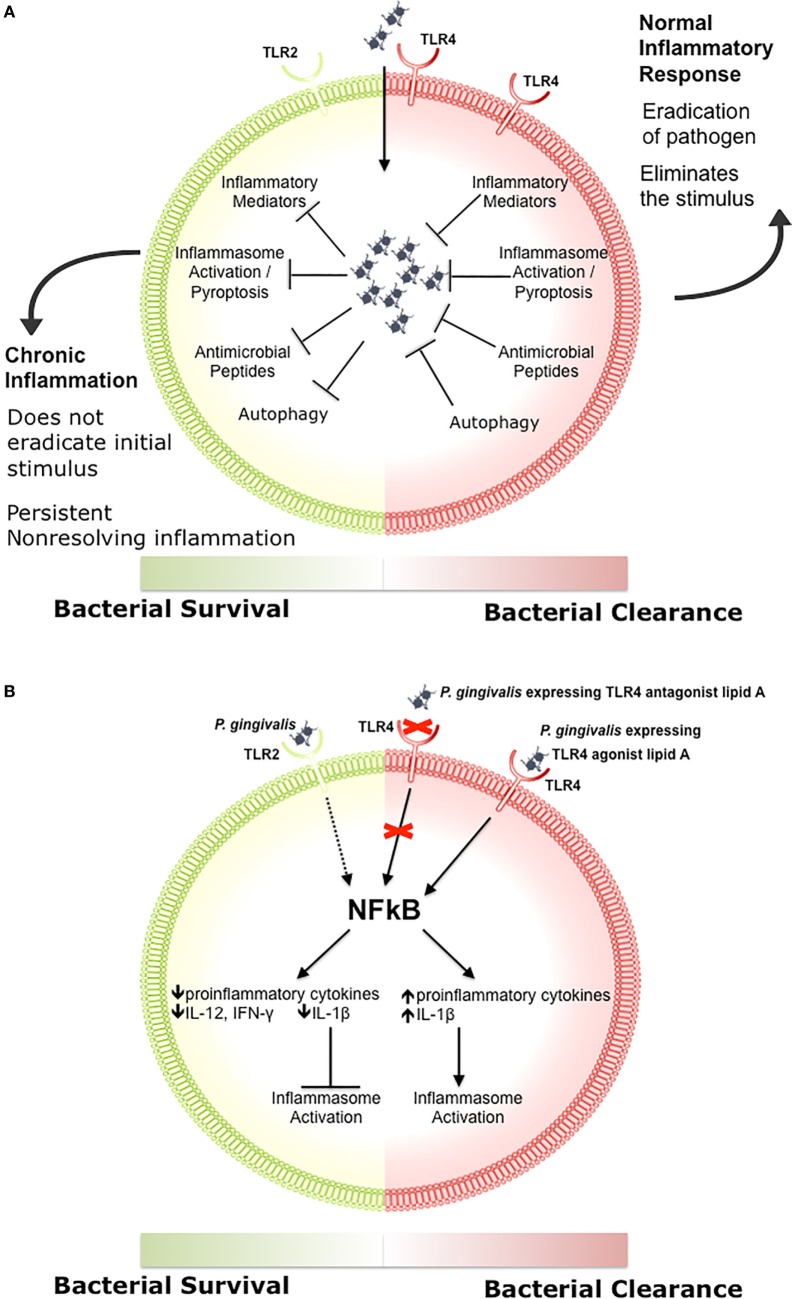
**The role of toll-like receptor (TLR) signaling in chronic inflammation**. **(A)** During a normal inflammatory response, activation of TLR signaling results in an increase in proinflammatory mediators and antimicrobial peptides, activation of the inflammasome, and clearance of the pathogen (97, 103, 104). Eradication of the stimulus results in resolution of inflammation (100–102). Some bacteria inhibit one or more of these responses, preventing the resolution of inflammation. **(B)** *Porphyromonas gingivalis* activation of TLR2 results in decreased production of proinflammatory cytokines such as IL-12 and IFN-γ, impairing bacterial clearance (109). *P. gingivalis* expressing a TLR4 antagonist lipid A moiety produces low levels of IL-1β and prevents activation of the non-canonical inflammasome, which also impairs bacterial clearance (108). In contrast, *P. gingivalis* expressing a TLR4 agonist lipid A moiety produces high levels of IL-1β and activates the inflammasome (108).

Engagement of TLR signaling triggers an inflammatory response that is primarily aimed at eliminating the invading organisms, initiating repair to damaged tissues, and initiating the adaptive immune response (83, 84, 86, 97–99). When well controlled, this beneficial inflammatory response manages a delicate balance between the clearance of pathogens and damage to the host through feedback loops and negative regulation, resolving once the stimulus has been removed (Figure 1A) (100–102). Clearance of pathogens is orchestrated through a combination of antimicrobial peptides, inflammatory mediators, phagocytosis, autophagy, and inflammasome activation (Figure 1A) (97, 103, 104). Chronic inflammation occurs when there is a breakdown in the regulation of these processes, which disrupts host cells locally and systemically, and it is increasingly associated with chronic conditions such as autoimmune diseases, cancer, IBD, arthritis, and atherosclerosis (105–107). We recently demonstrated that differential TLR signaling by variant *P. gingivalis* lipid A moieties was associated with the production of proinflammatory mediators and bacterial survival in macrophages (Figure 1B) (108).

A number of studies have examined the role of TLR signaling in microbial-induced chronic inflammation and immunopathology (70, 73, 76, 82). *Mycobacterium tuberculosis* and commensal gut microbiota have been shown to induce autoimmune arthritis through TLR signaling (73, 76). *E. coli* has been shown to increase non-small cell lung cancer metastasis in a TLR4-dependent manner (82). *Chlamydia pneumoniae* accelerates atherosclerosis through a TLR/MyD88-dependent mechanism (70). Our work has focused on defining the role of TLR2 and TLR4 signaling in *P. gingivalis*-mediated inflammatory atherosclerosis using a well-defined hyperlipidemic mouse model (ApoE^−/−^) (77–81). Numerous studies have documented a role for TLR signaling in lipid-induced atherosclerosis progression (110–113). Oxidized LDL particles are recruited to the atheroma and trigger TLR signaling in macrophages and endothelial cells. This results in foam cell formation and the production of proinflammatory cytokines and other proinflammatory mediators, such as IL-1, TNFα, and macrophage colony-stimulating factor, which perpetuate inflammation within the vasculature (109, 114, 115). It has been postulated that the association between microbial infection and atherosclerosis involves common mechanisms of signaling *via* TLR2 and TLR4. Some investigators have proposed that TLR signaling induced by multiple pathogens and endogenous ligands may explain the link to atherosclerosis and that therapeutic TLR antagonism could prove beneficial in the treatment of chronic atherosclerosis (13, 14, 116–118). However, our work revealed that *P. gingivalis*-mediated TLR4 signaling protects from atherosclerosis, suggesting that effects are pathogen specific.

We established that oral infection with *P. gingivalis* is associated with lipid accumulation and macrophage infiltration in the aortic sinus and innominate artery of ApoE^−/−^ mice (69, 71). *P. gingivalis* oral infection induced the expression of inflammatory mediators and proinflammatory cytokines such IFN-γ, IL-1β, interleukin-6 (IL-6), and TNFα in the atherosclerotic lesions of ApoE^−/−^ mice, which was significantly reduced in the atherosclerotic lesions of *P. gingivalis*-infected ApoE^−/−^TLR2^−/−^ mice (77–79, 81). In contrast to the effect of TLR2-deficiency on *P. gingivalis*-induced atherosclerosis, we demonstrate that TLR4-deficiency leads to increased disease severity, indicating a protective role for TLR4 signaling in *P. gingivalis-*induced atherosclerosis (80). *P. gingivalis*-infected TLR4-deficient mice had increased vascular inflammation characterized by enhanced lesion progression and increased macrophage accumulation compared to infected TLR4-sufficient mice (80). In contrast to what was observed with *P. gingivalis*, other reports have documented that TLR4-deficient mice infected intranasally with *C. pneumoniae* had diminished vascular inflammation compared to infected TLR4^+/+^ mice. These results suggest that the role of TLR4 signaling in atherosclerosis is pathogen specific.

*Porphyromonas gingivalis*-infected TLR4-deficient mice also had increased CD4/CD8 T cells, decreased regulatory T cell infiltration, and impaired Th1 immunity, implicating modulation of the adaptive immune response (80). Our results suggest that this protective role of TLR4 signaling may be orchestrated by dendritic cell (DC) IL-10 and IL-12 as well as by the induction of regulatory T cells (80). Collectively, our studies indicate that *P. gingivalis*-mediated dysregulation of innate and adaptive responses results in systemic inflammation and immunopathology.

## Innate Immune Subversion

A common theme that has recently emerged is that pathogens that are associated with chronic low-grade inflammation have developed mechanisms of immune subversion that either alter or inhibit protective signaling normally induced by TLRs. Lipid A, the biologically active moiety of LPS, can be expressed in variant forms by many human pathogens, allowing for evasion of the host innate immune system and establishment of a chronic infection (119–126). Table 1 summarizes the impact of impaired TLR4 signaling as a result of divergent lipid A of several immune subversive Gram-negative bacteria. Strikingly, several of these host-adapted Gram-negative bacteria that express immune-evasive lipid A are associated with increased risk of autoimmune disease, atherosclerosis, and cancer. Although *Helicobacter pylori* expresses a hexa-acylated species of lipid A, its predominant lipid A species is mono-phosphorylated and tetra-acylated; this combination of underphosphorylation and underacylation of lipid A likely explains the low endotoxic and biological activities of *H. pylori* LPS (119).

**Table 1:** **Gram-negative bacteria that express divergent lipid A structures**.

Bacterial strain	Acylation/phosphorylation	Toll-like receptor 4 activation	Outcomes	Reference
*Helicobacter pylori*	Mono-phosphorylated, tetra-acylated	Weak agonist	Bacterial survival	(119, 127)
*Pseudomonas aeruginosa*	Hepta-acylated	Strong agonist	Severe cystic fibrosis (CF), neutrophil survival	(120)
	Penta-acylated	Weak agonist	Decreased IL-8, CF	
*Bacteroides thetaiotaomicron*	Under-phosphorylated, penta-acylated	Weak agonist	Bacterial survival	(121, 128)
*Porphyromonas gingivalis*	Di-phosphorylated, penta-acylated	Agonist	Modest inflammation, decreased atherosclerosis	(108, 122)
	Mono-phosphorylated, tetra-acetylated	Antagonist	Systemic inflammation, increased atherosclerosis	
*S. flexneri*	Tri- or tetra-acylated	Weak agonist	Low cytokine production, inflammasome inhibition	(123)
*Neisseria meningitidis*	Penta-acylated	Non-activating	Low NFkB activation	(124)
*Yersinia pestis*	Hexa-acylated	Strong agonist	Bacterial clearance, no systemic disease	(125, 129, 130)
	Tetra-acylated (37°C)	Weak agonist	Systemic disease	
*Francisella tularensis*	Mono-phosphorylated, tetra-acetylated	Weak agonist	Decreased TNFα, bacterial survival	(126, 131, 132)

SenGupta et al. (120) reported that structural lipid A variants of *Pseudomonas aeruginosa* correlate with severity and progression of CF. Specifically, hepta-acylated lipid A is uniquely associated with severe late stage CF, and this variant acts as a strong TLR4 agonist, resulting in neutrophil survival and substantial production of IL-8 (120). Penta-acylated lipid A is found in patients with early stage or less severe (CF), accompanied by lower levels of IL-8 compared to those with severe late stage CF (120).

*Bacteroides thetaiotaomicron* expresses a penta-acylated 4′-dephosphorylated lipid A structure and exhibits resistance to various inflammation-associated CAMPs (121, 128). Using wild-type and *lpxF* mutant strains of *B. thetaiotaomicron*, Cullen et al. (128) demonstrated that CAMP resistance is LpxF-dependent but is also inflammation-dependent as *lpxF* deletion mutants were outcompeted by wild-type bacteria in germ-free mice only in the presence of inflammatory *Citrobacter rodentium* infection. These authors noted that LpxF orthologs have been identified in all sequenced human-associated Bacteroidetes. In addition to *B. thetaiotaomicron*, four other human-associated *Bacteroides* (*Bacteroides fragilis, Bacteroides vulgatus, Prevotella salivae, and P. gingivalis*) produce an under-phosphorylated lipid A structure (128).

Several Gram-negative bacteria that express immune-evasive lipid A species are associated with an increased risk of atherosclerosis (133, 134), but the oral pathogen *P. gingivalis* is a striking example of how lipid A variants allow a bacterium to evade TLR4 and promote chronic inflammation through dysregulation of both innate and adaptive immune responses (71, 78–80, 135).

In response to environmental conditions, *P. gingivalis* expresses a variety of lipid A species that are described as TLR4 agonist, antagonist, or non-stimulating depending on their phosphorylation and acylation states and the resulting immunostimulatory activity (122, 136–140). The underacylated lipid A moieties are poorly recognized by TLR4, and the antagonist lipid A species inhibits activation of TLR4 by agonist lipid A species from *P. gingivalis* or other Gram-negative organisms (122, 139, 141, 142). We recently examined the role of these *P. gingivalis* lipid A variants *in vitro* and in a mouse model of vascular inflammation. We demonstrated that the antagonist lipid A species enhanced bacterial survival in macrophages through inhibition of non-canonical inflammasome activation and decreased production of proinflammatory mediators such as IL-1β (Figure 1B) (108). In contrast, the agonist lipid A species was associated with decreased bacterial survival in macrophages and high levels of IL-1β (Figure 1B) (108). ApoE^−/−^ mice orally infected with the *P. gingivalis* wild-type or antagonist strains had progressive vascular inflammation characterized by enhanced lesion progression and increased macrophage accumulation compared to either sham-infected ApoE^−/−^ mice or ApoE^−/−^ mice that were orally infected with the *P. gingivalis* agonist strain. These studies indicate that distinct lipid A moieties allow *P. gingivalis* to evade the host innate immune response resulting in vascular inflammation. In addition to facilitating bacterial survival, it is possible that distinct lipid A moieties dysregulate host adaptive immune responses through manipulation of DC activation and downstream T cell polarization leading to systemic inflammatory pathologies.

## Dysregulation of Adaptive Immunity and Chronic Inflammation

Dysregulation of host immunity has been proposed to contribute to chronic inflammation observed in systemic inflammatory diseases and typically involves modulation of DC responses, critical antigen-presenting cells that link innate and adaptive immunity. DCs play a key role in immune-modulatory functions and are often targeted by pathogens, resulting in altered T cell-mediated adaptive responses, immune dysregulation, and immunopathology. Immature and highly phagocytic DCs reside in the tissues and detect pathogens through PRRs including DC-SIGN, TLR2, TLR4, NOD2, and the mannose receptor (143). PRR ligation and pathogen phagocytosis initiate DC maturation, which is characterized by a decrease in phagocytic capability and an upregulation of co-stimulatory molecules that are involved in activating T cells. The type of T cell response is dictated by which PRR is activated and, consequently, which cytokines are produced (143). The induction of a CD4 T cell response is largely determined by pathogen detection by DCs that present antigens to naïve T cells to initiate an adaptive response. DCs can also be activated to efficiently cross-present extracellular antigens on MHC-I, leading to activation of CD8 T cells (144, 145).

Lipopolysaccharide isolated from the Gram-negative pathogens *E. coli* and *Salmonella enterica* serotype Typhimurium potently stimulate TLR4 on DCs leading to maturation and expression of proinflammatory cytokines that drive a Th1 response (143). The expression of immune-evasive LPS by host-adapted bacteria such as *P. gingivalis* may result in different DC responses leading to altered T cell responses. However, the impact of immune-evasive LPS on systemic immunopathology has not been explored. We postulate that altered DC activation by *P. gingivalis* lipid A mutant strains will result in dysregulation of adaptive immunity that leads to enhanced development and progression of atherosclerosis. Current studies are underway to define how modification of lipid A alters DC maturation and functional responses.

## Implications for Future Therapies

Studies demonstrating a protective role for TLR deficiency in inflammation have prompted many to pursue therapeutic TLR antagonism for combating systemic inflammatory and autoimmune diseases. These TLR antagonists include structural analogs of agonists, anti-TLR antibodies, and small molecule antagonists. One example of a structural analog is eritoran, a TLR4 antagonist that inhibits LPS-induced inflammation and improves survival in a mouse model of sepsis (146). The small molecule inhibitor TAK-242 inhibits TLR4 signaling by binding selectively to TLR4 and disrupting interaction with its adaptor molecules (147). Wang et al. (148) demonstrated that TAK-242 diminished the accumulation of DCs, lymphocytes, macrophages, and neutrophils and enhanced production of IL-6, IL-8, and TNF-α in a mouse model of cigarette smoke-induced pulmonary inflammation. It has been suggested that therapy with eritoran or TAK-242 may be most efficacious against bacteria expressing hexa-acylated lipid A structures, which act as strong TLR4 agonists and elicit local inflammation but typically result in bacterial clearance and no systemic disease (149). In contrast, bacteria that do not produce hexa-acylated lipid A elicit little or no TLR4-mediated local inflammation, which permits bacterial survival and dissemination and contributes to the development and progression of systemic diseases. Anti-TLR4 therapy has been suggested as a prophylactic for necrotizing enterocolitis (NEC), but a recent study demonstrated that secretions from the probiotic *Bifidobacterium longum* subspecies *infantis*, often used to treat NEC, attenuate IL-1β-induced IL-6 in a TLR4-dependent manner in a human fetal small intestinal epithelial cell line (H4 cells) and primary NEC enterocytes (150). Despite the promising results of using TLR4 antagonists to prevent inflammation in mice, our results and others suggest that the role of TLR4 signaling in the pathogenesis of chronic inflammatory diseases is pathogen specific and that TLR4 antagonism could encourage systemic inflammation and dissemination of certain pathogens resulting in unintended outcomes (80, 108, 150). In addition, the presence of comorbid conditions and the bacterial characteristics of both local and distant microbiota create a complex environment in which chronic disease develops and progresses. For these reasons, we advocate caution in the development and testing of TLR4 antagonists for the treatment of chronic inflammatory diseases induced by the microbiota.

## Author Contributions

All authors listed have made substantial, direct, and intellectual contribution to the work and approved it for publication.

## Conflict of Interest Statement

The authors declare that the research was conducted in the absence of any commercial or financial relationships that could be construed as a potential conflict of interest.

## References

[1] Belkaid Y Hand TW. Role of the microbiota in immunity and inflammation. Cell (2014) 157:121–41. 10.1016/j.cell.2014.03.011 24679531 PMC4056765

[2] Marshall BJ Warren JR. Unidentified curved bacilli in the stomach of patients with gastritis and peptic ulceration. Lancet (1984) 1:1311–5. 10.1016/S0140-6736(84)91816-6 6145023

[3] Frank DN St Amand AL Feldman RA Boedeker EC Harpaz N Pace NR. Molecular-phylogenetic characterization of microbial community imbalances in human inflammatory bowel diseases. Proc Natl Acad Sci U S A (2007) 104:13780–5. 10.1073/pnas.0706625104 17699621 PMC1959459

[4] Sears CL Pardoll DM Perspective: alpha-bugs, their microbial partners, and the link to colon cancer. J Infect Dis (2011) 203:306–11. 10.1093/jinfdis/jiq061 21208921 PMC3071114

[5] Foxman B Martin ET. Use of the microbiome in the practice of epidemiology: a primer on -omic technologies. Am J Epidemiol (2015) 182:1–8. 10.1093/aje/kwv102 26025238 PMC4498138

[6] Robinson CK Brotman R Ravel J. Intricacies of assessing the human microbiome in epidemiologic studies. Ann Epidemiol (2016) 26:311–21. 10.1016/j.annepidem.2016.04.005 27180112 PMC4892937

[7] Vogtmann E Goedert JJ. Epidemiologic studies of the human microbiome and cancer. Br J Cancer (2016) 114:237–42. 10.1038/bjc.2015.465 26730578 PMC4742587

[8] Human Microbiome Project Consortium. Structure, function and diversity of the healthy human microbiome. Nature (2012) 486:207–14. 10.1038/nature11234 22699609 PMC3564958

[9] Morgan XC Huttenhower C. Chapter 12: human microbiome analysis. PLoS Comput Biol (2012) 8:e1002808. 10.1371/journal.pcbi.1002808 23300406 PMC3531975

[10] Guarner F Malagelada JR. Gut flora in health and disease. Lancet (2003) 361:512–9. 10.1016/S0140-6736(03)12489-0 12583961

[11] Garrett WS Gordon JI Glimcher LH. Homeostasis and inflammation in the intestine. Cell (2010) 140:859–70. 10.1016/j.cell.2010.01.023 20303876 PMC2845719

[12] Caverly LJ Zhao J LiPuma JJ. Cystic fibrosis lung microbiome: opportunities to reconsider management of airway infection. Pediatr Pulmonol (2015) 50:S31–8. 10.1002/ppul.23243 26335953

[13] Li J Hao C Ren L Xiao Y Wang J Qin X. Data mining of lung microbiota in cystic fibrosis patients. PLoS One (2016) 11(10):e0164510. 10.1371/journal.pone.0164510 27741283 PMC5065158

[14] Li H Hong F Pan S Lei L Yan F. Silencing triggering receptors expressed on myeloid cells-1 impaired the inflammatory response to oxidized low-density lipoprotein in macrophages. Inflammation (2016) 39:199–208. 10.1007/s10753-015-0239-5 26277357

[15] Hu X Zhang Q Hua H Chen F Changes in the salivary microbiota of oral leukoplakia and oral cancer. Oral Oncol (2016) 56:e6–8. 10.1016/j.oraloncology.2016.03.007 27026576

[16] Schroeder BO Bäckhed F. Signals from the gut microbiota to distant organs in physiology and disease. Nat Med (2016) 22:1079–89. 10.1038/nm.4185 27711063

[17] Rosser EC Mauri C. A clinical update on the significance of the gut microbiota in systemic autoimmunity. J Autoimmun (2016) 74:85–93. 10.1016/j.jaut.2016.06.009 27481556

[18] Scher JU Ubeda C Equinda M Khanin R Buischi Y Viale A Periodontal disease and the oral microbiota in new-onset rheumatoid arthritis. Arthritis Rheum (2012) 64:3083–94. 10.1002/art.34539 22576262 PMC3428472

[19] Yan X Yang M Liu J Gao R Hu J Li J Discovery and validation of potential bacterial biomarkers for lung cancer. Am J Cancer Res (2015) 5:3111–22. 26693063 PMC4656734

[20] Fåk F Tremaroli V Bergström G Bäckhed F Oral microbiota in patients with atherosclerosis. Atherosclerosis (2015) 243:573–8. 10.1016/j.atherosclerosis.2015.10.097 26536303

[21] Zhang CZ Cheng XQ Li JY Zhang P Yi P Xu X Saliva in the diagnosis of diseases. Int J Oral Sci (2016) 8:133–7. 10.1038/ijos.2016.38 27585820 PMC5113094

[22] Forman D Newell DG Fullerton F Yarnell JW Stacey AR Wald N Association between infection with *Helicobacter pylori* and risk of gastric cancer: evidence from a prospective investigation. BMJ (1991) 302:1302–5. 10.1136/bmj.302.6788.1302 2059685 PMC1670011

[23] Shor A Kuo CC Patton DL. Detection of *Chlamydia pneumoniae* in coronary arterial fatty streaks and atheromatous plaques. S Afr Med J (1992) 82:158–61. 1519134

[24] Kuo CC Gown AM Benditt EP Grayston JT. Detection of *Chlamydia pneumoniae* in aortic lesions of atherosclerosis by immunocytochemical stain. Arterioscler Thromb (1993) 13:1501–4. 10.1161/01.ATV.13.10.1501 7691166

[25] Ebringer A Wilson C. HLA molecules, bacteria and autoimmunity. J Med Microbiol (2000) 49:305–11. 10.1099/0022-1317-49-4-305 10755623

[26] Farsak B Yildirir A Akyön Y Pinar A Oç M Böke E Detection of *Chlamydia pneumoniae* and *Helicobacter pylori* DNA in human atherosclerotic plaques by PCR. J Clin Microbiol (2000) 38:4408–11. 11101572 10.1128/jcm.38.12.4408-4411.2000 PMC87613

[27] Haraszthy VI Zambon JJ Trevisan M Zeid M Genco RJ. Identification of periodontal pathogens in atheromatous plaques. J Periodontol (2000) 71:1554–60. 10.1902/jop.2000.71.10.1554 11063387

[28] Beck JD Elter JR Heiss G Couper D Mauriello SM Offenbacher S. Relationship of periodontal disease to carotid artery intima-media wall thickness: the atherosclerosis risk in communities (ARIC) study. Arterioscler Thromb Vasc Biol (2001) 21:1816–22. 10.1161/hq1101.097803 11701471

[29] Pussinen PJ Jousilahti P Alfthan G Palosuo T Asikainen S Salomaa V. Antibodies to periodontal pathogens are associated with coronary heart disease. Arterioscler Thromb Vasc Biol (2003) 23:1250–4. 10.1161/01.ATV.0000072969.71452.87 12714435

[30] Taniguchi A Nishimura F Murayama Y Nagasaka S Fukushima M Sakai M *Porphyromonas gingivalis* infection is associated with carotid atherosclerosis in non-obese Japanese type 2 diabetic patients. Metabolism (2003) 52:142–5. 10.1053/meta.2003.50001 12601622

[31] Pussinen PJ Alfthan G Tuomilehto J Asikainen S Jousilahti P. High serum antibody levels to *Porphyromonas gingivalis* predict myocardial infarction. Eur J Cardiovasc Prev Rehabil (2004) 11:408–11. 10.1097/01.hjr.0000129745.38217.39 15616414

[32] Latsios G Saetta A Michalopoulos NV Agapitos E Patsouris E. Detection of cytomegalovirus, *Helicobacter pylori* and *Chlamydia pneumoniae* DNA in carotid atherosclerotic plaques by the polymerase chain reaction. Acta Cardiol (2004) 59:652–7. 10.2143/AC.59.6.2005249 15636450

[33] Kozarov EV Dorn BR Shelburne CE Dunn WA Jr Progulske-Fox A Human atherosclerotic plaque contains viable invasive *Actinobacillus actinomycetemcomitans* and *Porphyromonas gingivalis*. Arterioscler Thromb Vasc Biol (2005) 25:17–8. 10.1161/01.ATV.0000155018.67835.1a 15662025

[34] Kaplan M Yavuz SS Cinar B Koksal V Kut MS Yapici F Detection of *Chlamydia pneumoniae* and *Helicobacter pylori* in atherosclerotic plaques of carotid artery by polymerase chain reaction. Int J Infect Dis (2006) 10:116–23. 10.1016/j.ijid.2004.10.008 16183317

[35] Wegner N Wait R Sroka A Eick S Nguyen KA Lundberg K Peptidylarginine deiminase from *Porphyromonas gingivalis* citrullinates human fibrinogen and α-enolase: implications for autoimmunity in rheumatoid arthritis. Arthritis Rheum (2010) 62:2662–72. 10.1002/art.27552 20506214 PMC2941529

[36] Castellarin M Warren RL Freeman JD Dreolini L Krzywinski M Strauss J *Fusobacterium nucleatum* infection is prevalent in human colorectal carcinoma. Genome Res (2012) 22:299–306. 10.1101/gr.126516.111 22009989 PMC3266037

[37] Michaud DS Izard J Wilhelm-Benartzi CS You DH Grote VA Tjønneland A Plasma antibodies to oral bacteria and risk of pancreatic cancer in a large European prospective cohort study. Gut (2013) 62:1764–70. 10.1136/gutjnl-2012-303006 22990306 PMC3815505

[38] Smyk DS Koutsoumpas AL Mytilinaiou MG Rigopoulou EI Sakkas LI Bogdanos DP. *Helicobacter pylori* and autoimmune disease: cause or bystander. World J Gastroenterol (2014) 20:613–29. 10.3748/wjg.v20.i3.613 24574735 PMC3921471

[39] Stagi S Rigante D Lepri G Bertini F Matucci-Cerinic M Falcini F. Evaluation of autoimmune phenomena in patients with pediatric autoimmune neuropsychiatric disorders associated with streptococcal infections (PANDAS). Autoimmun Rev (2014) 13:1236–40. 10.1016/j.autrev.2014.08.009 25151976

[40] Zhu H Shen Z Luo H Zhang W Zhu X. *Chlamydia trachomatis* infection-associated risk of cervical cancer: a meta-analysis. Medicine (Baltimore) (2016) 95:e3077. 10.1097/MD.0000000000003077 27043670 PMC4998531

[41] Manichanh C Rigottier-Gois L Bonnaud E Gloux K Pelletier E Frangeul L Reduced diversity of faecal microbiota in Crohn’s disease revealed by a metagenomic approach. Gut (2006) 55:205–11. 10.1136/gut.2005.073817 16188921 PMC1856500

[42] Dicksved J Halfvarson J Rosenquist M Järnerot G Tysk C Apajalahti J Molecular analysis of the gut microbiota of identical twins with Crohn’s disease. ISME J (2008) 2:716–27. 10.1038/ismej.2008.37 18401439

[43] Turnbaugh PJ Hamady M Yatsunenko T Cantarel BL Duncan A Ley RE A core gut microbiome in obese and lean twins. Nature (2009) 457:480–4. 10.1038/nature07540 19043404 PMC2677729

[44] Sokol H Seksik P. The intestinal microbiota in inflammatory bowel diseases: time to connect with the host. Curr Opin Gastroenterol (2010) 26:327–31. 10.1097/MOG.0b013e328339536b 20445446

[45] Giongo A Gano KA Crabb DB Mukherjee N Novelo LL Casella G Toward defining the autoimmune microbiome for type 1 diabetes. ISME J (2011) 5:82–91. 10.1038/ismej.2010.92 20613793 PMC3105672

[46] Kostic AD Xavier RJ Gevers D. The microbiome in inflammatory bowel disease: current status and the future ahead. Gastroenterology (2014) 146:1489–99. 10.1053/j.gastro.2014.02.009 24560869 PMC4034132

[47] Chen J Wright K Davis JM Jeraldo P Marietta EV Murray J An expansion of rare lineage intestinal microbes characterizes rheumatoid arthritis. Genome Med (2016) 8:43. 10.1186/s13073-016-0299-7 27102666 PMC4840970

[48] Scher JU Joshua V Artacho A Abdollahi-Roodsaz S Öckinger J Kullberg S The lung microbiota in early rheumatoid arthritis and autoimmunity. Microbiome (2016) 4:60 10.1186/s40168-016-0206-x 27855721 PMC5114783

[49] Willing BP Dicksved J Halfvarson J Andersson AF Lucio M Zheng Z A pyrosequencing study in twins shows that gastrointestinal microbial profiles vary with inflammatory bowel disease phenotypes. Gastroenterology (2010) 139:1844–54.e1. 10.1053/j.gastro.2010.08.049 20816835

[50] Joossens M Huys G Cnockaert M De Preter V Verbeke K Rutgeerts P. Dysbiosis of the faecal microbiota in patients with Crohn’s disease and their unaffected relatives. Gut (2011) 60:631–7. 10.1136/gut.2010.223263 21209126

[51] Negroni A Costanzo M Vitali R Superti F Bertuccini L Tinari A Characterization of adherent-invasive *Escherichia coli* isolated from pediatric patients with inflammatory bowel disease. Inflamm Bowel Dis (2012) 18:913–24. 10.1002/ibd.21899 21994005

[52] Machiels K Joossens M Sabino J De Preter V Arijs I Eeckhaut V A decrease of the butyrate-producing species *Roseburia hominis* and *Faecalibacterium prausnitzii* defines dysbiosis in patients with ulcerative colitis. Gut (2014) 63:1275–83. 10.1136/gutjnl-2013-304833 24021287

[53] Shah R Cope JL Nagy-Szakal D Dowd S Versalovic J Hollister EB Composition and function of the pediatric colonic mucosal microbiome in untreated patients with ulcerative colitis. Gut Microbes (2016) 7:384–96. 10.1080/19490976.2016.1190073 27217061 PMC5046168

[54] Takahashi K Nishida A Fujimoto T Fujii M Shioya M Imaeda H Reduced abundance of butyrate-producing bacteria species in the fecal microbial community in Crohn’s disease. Digestion (2016) 93:59–65. 10.1159/000441768 26789999

[55] Imhann F Vich Vila A Bonder MJ Fu J Gevers D Visschedijk MC Interplay of host genetics and gut microbiota underlying the onset and clinical presentation of inflammatory bowel disease. Gut (2016). 10.1136/gutjnl-2016-312135 27802154 PMC5699972

[56] Coleman OI Nunes T. Role of the microbiota in colorectal cancer: updates on microbial associations and therapeutic implications. Biores Open Access (2016) 5:279–88. 10.1089/biores.2016.0028 27790385 PMC5076480

[57] Pushalkar S Ji X Li Y Estilo C Yegnanarayana R Singh B Comparison of oral microbiota in tumor and non-tumor tissues of patients with oral squamous cell carcinoma. BMC Microbiol (2012) 12:144. 10.1186/1471-2180-12-144 22817758 PMC3507910

[58] Walther-António MR Chen J Multinu F Hokenstad A Distad TJ Cheek EH Potential contribution of the uterine microbiome in the development of endometrial cancer. Genome Med (2016) 8:122. 10.1186/s13073-016-0368-y 27884207 PMC5123330

[59] Ley RE Turnbaugh PJ Klein S Gordon JI. Microbial ecology: human gut microbes associated with obesity. Nature (2006) 444:1022–3. 10.1038/4441022a 17183309

[60] Brown CT Davis-Richardson AG Giongo A Gano KA Crabb DB Mukherjee N Gut microbiome metagenomics analysis suggests a functional model for the development of autoimmunity for type 1 diabetes. PLoS One (2011) 6:e25792. 10.1371/journal.pone.0025792 22043294 PMC3197175

[61] Murri M Leiva I Gomez-Zumaquero JM Tinahones FJ Cardona F Soriguer F Gut microbiota in children with type 1 diabetes differs from that in healthy children: a case-control study. BMC Med (2013) 11:46. 10.1186/1741-7015-11-46 23433344 PMC3621820

[62] de Goffau MC Luopajärvi K Knip M Ilonen J Ruohtula T Härkönen T Fecal microbiota composition differs between children with β-cell autoimmunity and those without. Diabetes (2013) 62:1238–44. 10.2337/db12-0526 23274889 PMC3609581

[63] Scher JU Sczesnak A Longman RS Segata N Ubeda C Bielski C Expansion of intestinal *Prevotella copri* correlates with enhanced susceptibility to arthritis. Elife (2013) 2:e01202. 10.7554/eLife.01202 24192039 PMC3816614

[64] Zhang X Zhang D Jia H Feng Q Wang D Liang D The oral and gut microbiomes are perturbed in rheumatoid arthritis and partly normalized after treatment. Nat Med (2015) 21:895–905. 10.1038/nm.3914 26214836

[65] Fan X Alekseyenko AV Wu J Peters BA Jacobs EJ Gapstur SM Human oral microbiome and prospective risk for pancreatic cancer: a population-based nested case-control study. Gut (2016). 10.1136/gutjnl-2016-312580 27742762 PMC5607064

[66] Yin J Liao SX He Y Wang S Xia GH Liu FT Dysbiosis of gut microbiota with reduced trimethylamine-N-oxide level in patients with large-artery atherosclerotic stroke or transient ischemic attack. J Am Heart Assoc (2015) 4:e002699. 10.1161/JAHA.115.002699 26597155 PMC4845212

[67] Moazed TC Campbell LA Rosenfeld ME Grayston JT Kuo CC. *Chlamydia pneumoniae* infection accelerates the progression of atherosclerosis in apolipoprotein E-deficient mice. J Infect Dis (1999) 180:238–41. 10.1086/314855 10353889

[68] Lalla E Lamster IB Hofmann MA Bucciarelli L Jerud AP Tucler S Oral infection with a periodontal pathogen accelerates early atherosclerosis in apolipoprotein E-null mice. Arterioscler Thromb Vasc Biol (2003) 23:1405–11. 10.1161/01.ATV.0000082462.26258.FE 12816879

[69] Miyamoto T Yumoto H Takahashi Y Davey M Gibson FC III Genco CA. Pathogen-accelerated atherosclerosis occurs early after exposure and can be prevented via immunization. Infect Immun (2006) 74:1376–80. 10.1128/IAI.74.2.1376-1380.2006 16428788 PMC1360301

[70] Naiki Y Sorrentino R Wong MH Michelsen KS Shimada K Chen S TLR/MyD88 and liver X receptor alpha signaling pathways reciprocally control *Chlamydia pneumoniae*-induced acceleration of atherosclerosis. J Immunol (2008) 181:7176–85. 10.4049/jimmunol.181.10.7176 18981139 PMC2683843

[71] Hayashi C Viereck J Hua N Phinikaridou A Madrigal AG Gibson FC III *Porphyromonas gingivalis* accelerates inflammatory atherosclerosis in the innominate artery of ApoE deficient mice. Atherosclerosis (2011) 215:52–9. 10.1016/j.atherosclerosis.2010.12.009 21251656 PMC3057233

[72] Marchesan JT Gerow EA Schaff R Taut AD Shin SY Sugai J *Porphyromonas gingivalis* oral infection exacerbates the development and severity of collagen-induced arthritis. Arthritis Res Ther (2013) 15:R186. 10.1186/ar4376 24456966 PMC3979094

[73] Kanagawa H Niki Y Kobayashi T Sato Y Katsuyama E Fujie A *Mycobacterium tuberculosis* promotes arthritis development through toll-like receptor 2. J Bone Miner Metab (2015) 33:135–41. 10.1007/s00774-014-0575-9 24633489

[74] Lakritz JR Poutahidis T Mirabal S Varian BJ Levkovich T Ibrahim YM Gut bacteria require neutrophils to promote mammary tumorigenesis. Oncotarget (2015) 6:9387–96. 10.18632/oncotarget.3328 25831236 PMC4496224

[75] Sorrentino R Yilmaz A Schubert K Crother TR Pinto A Shimada K A single infection with *Chlamydia pneumoniae* is sufficient to exacerbate atherosclerosis in ApoE deficient mice. Cell Immunol (2015) 294:25–32. 10.1016/j.cellimm.2015.01.007 25666507 PMC4391498

[76] Abdollahi-Roodsaz S Joosten LA Helsen MM Walgreen B van Lent PL van den Bersselaar LA Shift from toll-like receptor 2 (TLR-2) toward TLR-4 dependency in the erosive stage of chronic streptococcal cell wall arthritis coincident with TLR-4-mediated interleukin-17 production. Arthritis Rheum (2008) 58:3753–64. 10.1002/art.24127 19035506

[77] Liu X Ukai T Yumoto H Davey M Goswami S Gibson FC III Toll-like receptor 2 plays a critical role in the progression of atherosclerosis that is independent of dietary lipids. Atherosclerosis (2008) 196:146–54. 10.1016/j.atherosclerosis.2007.03.025 17466307 PMC2243224

[78] Hayashi C Madrigal AG Liu X Ukai T Goswami S Gudino CV Pathogen-mediated inflammatory atherosclerosis is mediated in part via toll-like receptor 2-induced inflammatory responses. J Innate Immun (2010) 2:334–43. 10.1159/000314686 20505314 PMC2895755

[79] Hayashi C Gudino CV Gibson FC III Genco CA. Review: pathogen-induced inflammation at sites distant from oral infection: bacterial persistence and induction of cell-specific innate immune inflammatory pathways. Mol Oral Microbiol (2010) 25:305–16. 10.1111/j.2041-1014.2010.00582.x 20883220 PMC2951292

[80] Hayashi C Papadopoulos G Gudino CV Weinberg EO Barth KR Madrigal AG Protective role for TLR4 signaling in atherosclerosis progression as revealed by infection with a common oral pathogen. J Immunol (2012) 189:3681–8. 10.4049/jimmunol.1201541 22956579 PMC3448820

[81] Papadopoulos G Weinberg EO Massari P Gibson FC III Wetzler LM Morgan EF Macrophage-specific TLR2 signaling mediates pathogen-induced TNF-dependent inflammatory oral bone loss. J Immunol (2013) 190:1148–57. 10.4049/jimmunol.1202511 23264656 PMC3549226

[82] Chow SC Gowing SD Cools-Lartigue JJ Chen CB Berube J Yoon HW Gram negative bacteria increase non-small cell lung cancer metastasis via toll-like receptor 4 activation and mitogen-activated protein kinase phosphorylation. Int J Cancer (2015) 136:1341–50. 10.1002/ijc.29111 25082668

[83] Janeway CA Jr Approaching the asymptote? Evolution and revolution in immunology. Cold Spring Harb Symp Quant Biol (1989) 54(Pt 1):1–13. 10.1101/SQB.1989.054.01.003 2700931

[84] Blasius AL Beutler B. Intracellular toll-like receptors. Immunity (2010) 32:305–15. 10.1016/j.immuni.2010.03.012 20346772

[85] Aliprantis AO Yang RB Mark MR Suggett S Devaux B Radolf JD Cell activation and apoptosis by bacterial lipoproteins through toll-like receptor-2. Science (1999) 285:736–9. 10.1126/science.285.5428.736 10426996

[86] Brightbill HD Libraty DH Krutzik SR Yang RB Belisle JT Bleharski JR Host defense mechanisms triggered by microbial lipoproteins through toll-like receptors. Science (1999) 285:732–6. 10.1126/science.285.5428.732 10426995

[87] Schwandner R Dziarski R Wesche H Rothe M Kirschning CJ. Peptidoglycan- and lipoteichoic acid-induced cell activation is mediated by toll-like receptor 2. J Biol Chem (1999) 274:17406–9. 10.1074/jbc.274.25.17406 10364168

[88] Underhill DM Ozinsky A Hajjar AM Stevens A Wilson CB Bassetti M The toll-like receptor 2 is recruited to macrophage phagosomes and discriminates between pathogens. Nature (1999) 401:811–5. 10.1038/44605 10548109

[89] Massari P Visintin A Gunawardana J Halmen KA King CA Golenbock DT Meningococcal porin PorB binds to TLR2 and requires TLR1 for signaling. J Immunol (2006) 176:2373–80. 10.4049/jimmunol.176.4.2373 16455995

[90] Poltorak A He X Smirnova I Liu MY Van Huffel C Du X Defective LPS signaling in C3H/HeJ and C57BL/10ScCr mice: mutations in Tlr4 gene. Science (1998) 282:2085–8. 10.1126/science.282.5396.2085 9851930

[91] Qureshi ST Larivière L Leveque G Clermont S Moore KJ Gros P Endotoxin-tolerant mice have mutations in toll-like receptor 4 (Tlr4). J Exp Med (1999) 189:615–25. Erratum in: *J Exp Med* (1999) 189:1518. 10.1084/jem.189.4.615 9989976 PMC2192941

[92] Hoshino K Takeuchi O Kawai T Sanjo H Ogawa T Takeda Y Cutting edge: toll-like receptor 4 (TLR4)-deficient mice are hyporesponsive to lipopolysaccharide: evidence for TLR4 as the Lps gene product. J Immunol (1999) 162:3749–52. 10201887

[93] Park BS Lee JO. Recognition of lipopolysaccharide pattern by TLR4 complexes. Exp Mol Med (2013) 45:e66. 10.1038/emm.2013.97 24310172 PMC3880462

[94] Takeda K Akira S TLR signaling pathways. Semin Immunol (2006) 16:3–9. 10.1016/j.smim.2003.10.003 14751757

[95] Kagan JC Su T Horng T Chow A Akira S Medzhitov R. TRAM couples endocytosis of toll-like receptor 4 to the induction of interferon-beta. Nat Immunol (2008) 9:361–8. 10.1038/ni1569 18297073 PMC4112825

[96] Seong SY Matzinger P Hydrophobicity: an ancient damage-associated molecular pattern that initiates innate immune responses. Nat Rev Immunol (2004) 4:469–78. 10.1038/nri1372 15173835

[97] Medzhitov R Janeway C Jr Innate immunity. N Engl J Med (2000) 343:338–44. 10.1056/NEJM200008033430506 10922424

[98] Akira S Takeda K Toll-like receptor signalling. Nat Rev Immunol (2004) 4:499–511. 10.1038/nri1391 15229469

[99] Akira S Uematsu S Takeuchi O Pathogen recognition and innate immunity. Cell (2006) 124:783–801. 10.1016/j.cell.2006.02.015 16497588

[100] Liew FY Xu D Brint EK O’Neill LA. Negative regulation of toll-like receptor-mediated immune responses. Nat Rev Immunol (2005) 5:446–58. 10.1038/nri1630 15928677

[101] Kluwe J Mencin A Schwabe RF. Toll-like receptors, wound healing, and carcinogenesis. J Mol Med (Berl) (2009) 87:125–38. 10.1007/s00109-008-0426-z 19089397 PMC2791674

[102] Ospelt C Gay S. TLRs and chronic inflammation. Int J Biochem Cell Biol (2010) 42:495–505. 10.1016/j.biocel.2009.10.010 19840864

[103] Becker CE O’Neill LA Inflammasomes in inflammatory disorders: the role of TLRs and their interactons with NLRs. Semin Immunopathol (2007) 29:239–48. 10.1007/s00281-007-0081-4 17805544

[104] Mogensen TH. Pathogen recognition and inflammatory signaling in innate immune defenses. Clin Microbiol Rev (2009) 22:240–73. 10.1128/CMR.00046-08 19366914 PMC2668232

[105] Firestein GS. Evolving concepts of rheumatoid arthritis. Nature (2003) 423:356–61. 10.1038/nature01661 12748655

[106] Hansson GK Libby P Tabas I. Inflammation and plaque vulnerability. J Intern Med (2015) 278:483–93. 10.1111/joim.12406 26260307 PMC5082111

[107] Axelrad JE Lichtiger S Yajnik V. Inflammatory bowel disease and cancer: the role of inflammation, immunosuppression, and cancer treatment. World J Gastroenterol (2016) 22:4794–801. 10.3748/wjg.v22.i20.4794 27239106 PMC4873872

[108] Slocum C Coats SR Hua N Kramer C Papadopoulos G Weinberg EO Distinct lipid a moieties contribute to pathogen-induced site-specific vascular inflammation. PLoS Pathog (2014) 10:e1004215. 10.1371/journal.ppat.1004215 25010102 PMC4092147

[109] Hajishengallis G Lambris JD. Microbial manipulation of receptor crosstalk in innate immunity. Nat Rev Immunol (2011) 11:187–200. 10.1038/nri2918 21350579 PMC3077082

[110] Bjorkbacka H Kunjathoor VV Moore KJ Koehn S Ordija CM Lee MA Reduced atherosclerosis in MyD88-null mice links elevated serum cholesterol levels to activation of innate immunity signaling pathways. Nat Med (2004) 10:416–21. 10.1038/nm1008 15034566

[111] Michelsen KS Wong MH Shah PK Zhang W Yano J Doherty TM Lack of toll-like receptor 4 or myeloid differentiation factor 88 reduces atherosclerosis and alters plaque phenotype in mice deficient in apolipoprotein E. Proc Natl Acad Sci U S A (2004) 101:10679–84. 10.1073/pnas.0403249101 15249654 PMC489994

[112] Curtiss LK Tobias PS. The toll of toll-like receptors, especially toll-like receptor 2, on murine atherosclerosis. Curr Drug Targets (2007) 8:1230–8. 10.2174/138945007783220605 18220700

[113] den Dekker WK Cheng C Pasterkamp G Duckers HJ. Toll like receptor 4 in atherosclerosis and plaque destabilization. Atherosclerosis (2010) 209:314–20. 10.1016/j.atherosclerosis.2009.09.075 19900676

[114] Ross R. The pathogenesis of atherosclerosis: a perspective for the 1990s. Nature (1993) 362:801–9. 10.1038/362801a0 8479518

[115] Feghali C Wright T Cytokines in acute and chronic inflammation. Front Biosci (1997) 2:12–26. 10.2741/A171 9159205

[116] Ishii KJ Uematsu S Akira S. ‘Toll’ gates for future immunotherapy. Curr Pharm Des (2006) 12:4135–42. 10.2174/138161206778743484 17100616

[117] Monaco C Gregan SM Navin TJ Foxwell BM Davies AH Feldmann M. Toll-like receptor-2 mediates inflammation and matrix degradation in human atherosclerosis. Circulation (2009) 120:2462–9. 10.1161/CIRCULATIONAHA.109.851881 19948979

[118] Lu Z Zhang X Li Y Lopes-Virella MF Huang Y. TLR4 antagonist attenuates atherogenesis in LDL receptor-deficient mice with diet-induced type 2 diabetes. Immunobiology (2015) 220:1246–54. 10.1016/j.imbio.2015.06.016 26162692 PMC4558266

[119] Moran AP Lindner B Walsh EJ Structural characterization of the lipid A component of *Helicobacter pylori* rough- and smooth-form lipopolysaccharides. J Bacteriol (1997) 179:6453–63. 10.1128/jb.179.20.6453-6463.1997 9335296 PMC179563

[120] SenGupta S Hittle LE Ernst RK Uriarte SM Mitchell TC. A *Pseudomonas aeruginosa* hepta-acylated lipid A variant associated with cystic fibrosis selectively activates human neutrophils. J Leukoc Biol (2016) 100:1047–59. 10.1189/jlb.4VMA0316-101R 27538572 PMC6608067

[121] Coats SR Berezow AB To TT Jain S Bainbridge BW Banani KP The lipid A phosphate position determines differential host toll-like receptor 4 responses to phylogenetically related symbiotic and pathogenic bacteria. Infect Immun (2011) 79:203–10. 10.1128/IAI.00937-10 20974832 PMC3019871

[122] Coats SR Jones JW Do CT Braham PH Bainbridge BW To TT Human toll-like receptor 4 responses to *P. gingivalis* are regulated by lipid A 1- and 4’-phosphatase activities. Cell Microbiol (2009) 11:1587–99. 10.1111/j.1462-5822.2009.01349.x 19552698 PMC3074576

[123] Paciello I Silipo A Lembo-Fazio L Curcurù L Zumsteg A Noël G Intracellular *Shigella* remodels its LPS to dampen the innate immune recognition and evade inflammasome activation. Proc Natl Acad Sci U S A (2013) 110:E4345–54. 10.1073/pnas.1303641110 24167293 PMC3832022

[124] Steeghs L Keestra AM van Mourik A Uronen-Hansson H van der Ley P Callard R Differential activation of human and mouse toll-like receptor 4 by the adjuvant candidate LpxL1 of *Neisseria meningitidis*. Infect Immun (2008) 76:3801–7. 10.1128/IAI.00005-08 18490457 PMC2493235

[125] Kawahara K Tsukano H Watanabe H Lindner B Matsuura M. Modification of the structure and activity of lipid A in *Yersinia pestis* lipopolysaccharide by growth temperature. Infect Immun (2002) 70:4092–8. 10.1128/IAI.70.8.4092-4098.2002 12117916 PMC128165

[126] Sandström G Sjöstedt A Johansson T Kuoppa K Williams JC. Immunogenicity and toxicity of lipopolysaccharide from *Francisella tularensis* LVS. FEMS Microbiol Immunol (1992) 5:201–10. 10.1111/j.1574-6968.1992.tb05902.x 1419118

[127] Cullen TW Giles DK Wolf LN Ecobichon C Boneca IG Trent MS *Helicobacter pylori* versus the host: remodeling of the bacterial outer membrane is required for survival in the gastric mucosa. PLoS Pathog (2011) 7:e1002454 10.1371/journal.ppat.1002454 22216004 PMC3245313

[128] Cullen TW Schofield WB Barry NA Putnam EE Rundell EA Trent MS Gut microbiota. Antimicrobial peptide resistance mediates resilience of prominent gut commensals during inflammation. Science (2015) 347:170–5. 10.1126/science.1260580 25574022 PMC4388331

[129] Rebeil R Ernst RK Gowen BB Miller SI Hinnebusch BJ. Variation in lipid A structure in the pathogenic yersiniae. Mol Microbiol (2004) 52:1363–73. 10.1111/j.1365-2958.2004.04059.x 15165239

[130] Montminy SW Khan N McGrath S Walkowicz MJ Sharp F Conlon JE Virulence factors of *Yersinia pestis* are overcome by a strong lipopolysaccharide response. Nat Immunol (2006) 7:1066–73. 10.1038/ni1386 16980981

[131] Vinogradov E Perry MB Conlan JW. Structural analysis of *Francisella tularensis* lipopolysaccharide. Eur J Biochem (2002) 269:6112–8. 10.1046/j.1432-1033.2002.03321.x 12473106

[132] Phillips NJ Schilling B McLendon MK Apicella MA Gibson BW. Novel modification of lipid A of *Francisella tularensis*. Infect Immun (2004) 72:5340–8. 10.1128/IAI.72.9.5340-5348.2004 15322031 PMC517411

[133] Ameriso SF Fridman EA Leiguarda RC Sevlever GE. Detection of *Helicobacter pylori* in human carotid atherosclerotic plaques. Stroke (2001) 32:385–91. 10.1161/01.STR.32.2.385 11157171

[134] Belland RJ Ouellette SP Gieffers J Byrne GI. *Chlamydia pneumoniae* and atherosclerosis. Cell Microbiol (2004) 6:117–27. 10.1046/j.1462-5822.2003.00352.x 14706098

[135] Barth K Remick DG Genco CA. Disruption of immune regulation by microbial pathogens and resulting chronic inflammation. J Cell Physiol (2013) 228:1413–22. 10.1002/jcp.24299 23255141 PMC3995356

[136] Tabeta K Yamazaki K Akashi S Miyake K Kumada H Umemoto T Toll-like receptors confer responsiveness to lipopolysaccharide from *Porphyromonas gingivalis* in human gingival fibroblasts. Infect Immun (2000) 68:3731–5. 10.1128/IAI.68.6.3731-3735.2000 10816537 PMC97668

[137] Yoshimura A Kaneko T Kato Y Golenbock DT Hara Y. Lipopolysaccharides from periodontopathic bacteria *Porphyromonas gingivalis* and *Capnocytophaga ochracea* are antagonists for human toll-like receptor 4. Infect Immun (2002) 70:218–25. 10.1128/IAI.70.1.218-225.2002 11748186 PMC127593

[138] Coats SR Reife RA Bainbridge BW Pham TT Darveau RP. *Porphyromonas gingivalis* lipopolysaccharide antagonizes *Escherichia coli* lipopolysaccharide at toll-like receptor 4 in human endothelial cells. Infect Immun (2003) 71:6799–807. 10.1128/IAI.71.12.6799-6807.2003 14638766 PMC308937

[139] Reife RA Coats SR Al-Qutub M Dixon DM Braham PA Billharz RJ *Porphyromonas gingivalis* lipopolysaccharide lipid A heterogeneity: differential activities of tetra- and penta-acylated lipid A structures on E-selectin expression and TLR4 recognition. Cell Microbiol (2006) 8:857–68. 10.1111/j.1462-5822.2005.00672.x 16611234

[140] Kumada H Haishima Y Watanabe K Hasegawa C Tsuchiya T Tanamoto K Biological properties of the native and synthetic lipid A of *Porphyromonas gingivalis* lipopolysaccharide. Oral Microbiol Immunol (2008) 23:60–9. 10.1111/j.1399-302X.2007.00392.x 18173800

[141] Bostanci N Allaker RP Belibasakis GN Rangarajan M Curtis MA Hughes FJ *Porphyromonas gingivalis* antagonises *Campylobacter rectus* induced cytokine production by human monocytes. Cytokine (2007) 39:147–56. 10.1016/j.cyto.2007.07.002 17709256

[142] Coats SR Do CT Karimi-Naser LM Braham PH Darveau RP. Antagonistic lipopolysaccharides block *E. coli* lipopolysaccharide function at human TLR4 via interaction with the human MD-2 lipopolysaccharide binding site. Cell Microbiol (2007) 9:1191–202. 10.1111/j.1462-5822.2006.00859.x 17217428

[143] Pulendran B. Variegation of the immune response with dendritic cells and pathogen recognition receptors. J Immunol (2005) 174:2457–65. 10.4049/jimmunol.174.5.2457 15728447

[144] Joffre OP Segura E Savina A Amigorena S. Cross-presentation by dendritic cells. Nat Rev Immunol (2012) 12:557–69. 10.1038/nri3254 22790179

[145] Alloatti A Kotsias F Pauwels AM Carpier JM Jouve M Timmerman E Toll-like receptor 4 engagement on dendritic cells restrains phago-lysosome fusion and promotes cross-presentation of antigens. Immunity (2015) 43:1087–100. 10.1016/j.immuni.2015.11.006 26682983

[146] Mullarkey M Rose JR Bristol J Kawata T Kimura A Kobayashi S Inhibition of endotoxin response by e5564, a novel toll-like receptor 4-directed endotoxin antagonist. J Pharmacol Exp Ther (2003) 304:1093–102. 10.1124/jpet.102.044487 12604686

[147] Matsunaga N Tsuchimori N Matsumoto T Ii M. TAK-242 (resatorvid), a small-molecule inhibitor of toll-like receptor (TLR) 4 signaling, binds selectively to TLR4 and interferes with interactions between TLR4 and its adaptor molecules. Mol Pharmacol (2011) 79:34–41. 10.1124/mol.110.068064 20881006

[148] Wang D Tao K Xion J Xu S Jiang Y Chen Q TAK-242 attenuates acute cigarette smoke-induced pulmonary inflammation in mouse via the TLR4/NF-κB signaling pathway. Biochem Biophys Res Commun (2016) 472:508–15. 10.1016/j.bbrc.2016.03.001 26944017

[149] Munford RS Sensing Gram-negative bacterial lipopolysaccharides: a human disease determinant? Infect Immun (2008) 76:454–65. 10.1128/IAI.00939-07 18086818 PMC2223455

[150] Meng D Zhu W Ganguli K Shi HN Walker WA. Anti-inflammatory effects of *Bifidobacterium longum* subsp *infantis* secretions on fetal human enterocytes are mediated by TLR-4 receptors. Am J Physiol Gastrointest Liver Physiol (2016) 311:G744–53. 10.1152/ajpgi.00090.2016 27562058 PMC5142200

